# Role of Gut Microbiome in Neoadjuvant Chemotherapy Response in Urothelial Carcinoma: A Multi-institutional Prospective Cohort Evaluation

**DOI:** 10.1158/2767-9764.CRC-23-0479

**Published:** 2024-06-17

**Authors:** Laura Bukavina, Rashida Ginwala, Mohamed Eltoukhi, Mohit Sindhani, Megan Prunty, Daniel M. Geynisman, Pooja Ghatalia, Henkel Valentine, Adam Calaway, Andres F. Correa, Jason R. Brown, Kirtishri Mishra, Elizabeth R. Plimack, Alexander Kutikov, Mahmoud Ghannoum, Mohammed Elshaer, Mauricio Retuerto, Lee Ponsky, Robert G. Uzzo, Philip H. Abbosh

**Affiliations:** 1 Fox Chase Cancer Center, Philadelphia, Pennsylvania.; 2 Cleveland Clinic Glickman Urologic Institute, Cleveland, Ohio.; 3 Case Western Reserve School of Medicine, Cleveland, Ohio.; 4 India Institute of Technology, Delhi, India.; 5 University Hospitals Cleveland Medical Center, Cleveland, Ohio.; 6 Albert Einstein Medical Center, Philadelphia, Pennsylvania.

## Abstract

Neoadjuvant chemotherapy (NAC) is linked with clinical advantages in urothelial carcinoma for patients with muscle-invasive bladder cancer (MIBC). Despite comprehensive research into the influence of tumor mutation expression profiles and clinicopathologic factors on chemotherapy response, the role of the gut microbiome (GM) in bladder cancer chemotherapy response remains poorly understood. This study examines the variance in the GM of patients with bladder cancer compared with healthy adults, and investigates GM compositional differences between patients who respond to chemotherapy versus those who exhibit residual disease.

Our study reveals distinct clustering, effectively separating the bladder cancer and healthy cohorts. However, no significant differences were observed between chemotherapy responders and nonresponders within community subgroups. Machine learning models based on responder status outperformed clinical variables in predicting complete response (AUC 0.88 vs. AUC 0.50), although no single microbial species emerged as a fully reliable biomarker.

The evaluation of short chain fatty acid (SCFA) concentration in blood and stool revealed no correlation with responder status. Still, SCFA analysis showed a higher abundance of Akkermansia (rs = 0.51, *P* = 0.017) and Clostridia (rs = 0.52, *P* = 0.018), which correlated with increased levels of detectable fecal isobutyric acid. Higher levels of fecal Lactobacillus (rs = 0.49, *P* = 0.02) and Enterobacteriaceae (rs = 0.52, *P* < 0.03) correlated with increased fecal propionic acid.

In conclusion, our study constitutes the first large-scale, multicenter assessment of GM composition, suggesting the potential for a complex microbial signature to predict patients more likely to respond to NAC based on multiple taxa.

**Significance:**

Our study highlights results that link the composition of the GM to the efficacy of NAC in MIBC. We discovered that patients with higher levels of Bacteroides experienced a worse response to NAC. This microbial signature shows promise as a superior predictor of treatment response over traditional clinical variables. Although preliminary, our findings advocate for larger, more detailed studies to validate these associations.

## Introduction

Treatment with neoadjuvant chemotherapy (NAC) in muscle-invasive bladder cancer (MIBC) is associated with clinical benefit in urothelial carcinoma. Cisplatin-based NAC prior to extirpative surgery confers a 6%–8% overall survival (OS) benefit compared with surgery alone (1). Historically, the two most commonly utilized regimens include dose-dense methotrexate, vinblastine, doxorubicin, and cisplatin (dd-MVAC) or gemcitabine and cisplatin (GC), with pathologic complete response (pT0) after NAC at the time of surgery being widely adopted as a surrogate endpoint for improved OS (2). Conversely, patients with residual disease or nodal involvement are at a high risk of recurrence and progression of disease.

While extensive research evaluating role of tumor mutational expression profiles and clinicopathologic factors into chemotherapy response has been published, the role of gut microbiome (GM) in bladder cancer in chemotherapy response has not been thoroughly evaluated. Indeed, the GM is increasingly seen as important and modifiable aspect of anticancer therapeutic response (3, 4). We hypothesized that GM composition associates with response to therapy in bladder cancer. To test this hypothesis, we prospectively collected fecal microbiome samples from patients with MIBC starting treatment with NAC across two American institutions. In addition, samples from partners living with patients during NAC were collected to identify changes caused by chemotherapy versus confounding environmental effects.

Furthermore, to investigate the change in GM during carcinogenesis using the N-butyl-N-(4-hydroxybutyl)-nitrosamine (BBN) murine model, stool pellets were collected for GM profiling throughout the BBN exposure period and at the time of sacrifice. This research provides the largest assessment of GM and its association with response to NAC and facilitates specific microbial special investigation and functions associated with response.

## Materials and Methods

### Patient Cohort and Sample Collection

The study was conducted in accordance with recognized ethical guidelines and approved by Case Western Reserve/University Hospitals Cleveland Medical Center (CW) and Fox Chase Cancer Center (FCCC) under the Institutional Review Board (IRB)# STUDY20200350 and IRB #18-4001, respectively. Patients with MIBC undergoing cystectomy were enrolled prospectively across both institutions, written informed consent was obtained.

Stool samples were collected prior to cystectomy between July, 2018 until March, 2023. Patients were excluded from participation if they have any antibiotics within 6 weeks of collection or history of *Clostridium difficile* infection and treatment within 2 months. All patients were > 6 weeks out from last cystoscopy requiring antibiotics or transurethral resection of bladder tumor. Presurgical stool samples were collected at the time of cystectomy prior to initiation of antibiotics with rectal swabs, and all collections were performed prior to the use of preoperative antibiotics for surgical preparation (Supplementary Fig. S1). Sample sizes were determined on the basis of availability of eligible participants and the feasibility of sample collection within the study timeline.

A total of 142 patients with bladder cancer were recruited for the study after meeting inclusion criteria (Supplementary Fig. S2). The swabs were immediately placed into 1.5 mL microcentrifuge tubes containing 1 mL of PBS. Swabs were stored at 0°C during transport to the laboratory for processing within 2 hours of collection. Samples were then resuspended and stored in sterile PBS at −80°C until analysis. Partners and changes during chemotherapy: stool samples were collected prior to NAC (dd-MVAC), after cycle 2 (of 3), and prior to cystectomy. Stool samples from partners were collected at the same time and stored. Samples were stored at room temperature for <24 hours prior to being split into aliquots and stored at 80°C until use. All patients and partners were provided an OMNIgene GUT kit (OMR-200; DNA Genotek) for outpatient fecal sample collection, which maintains DNA stability at room temperate for up to 60 days. The final analysis consisted of 190 patients (142 bladder cancer, 48 Controls) (Supplementary Appendix; Supplementary Table S1).

### Murine Studies

Specific pathogen-free C57BL/6 female and male mice were purchased from Taconic Laboratory. The animal experiments were carried out following FCCC Institutional Animal Care and Use Committee guideline and approved (IACUC 19-03). BBN was purchased as a single batch from TCI America (Batch ODW3F-FH). BBN was administered at a concentration of 0.05% in water given ad libitum and replenished weekly to half of the cages, with the other half receiving regular drinking water from the same source. Half of the mice in each treatment arm were male. Mice began BBN administration via drinking water at 8–10 weeks of age and continued for 12 weeks. After 12 weeks of BBN exposure, mice were given regular drinking water until the end of the study. Presence of tumors was evaluated via excretory µCT urography following retroorbital injection of dilute Visipaque contrast (Supplementary Fig. S3). Stool pellets were collected at pretreatment, 6 weeks, 12 weeks, and between 16 to 22 weeks (when tumors were visible as filling defects on µCT scan). A total of 23 mice were exposed to BBN (14 males, 9 females), with 18 water only controls (10 males, 8 females; Supplementary Fig. S3). When sacrificing mice, one mouse from each cage was sacrificed at each timepoint rather than sacrificing all mice from one cage to avoid confounding factors that might be present in one cage and not another. Stool pellets were collected from each mouse if it spontaneously defecated or with gentle abdominal pressure. Pellets were resuspended in PBS and stored −80 °C until use.

### DNA Extraction and Bacterial 16S rRNA Sequencing

Fecal samples were stored in 250 µL aliquots at −80°C for DNA extraction. DNA was extracted from the thawed fecal samples using the DNeasy blood and tissue kit (Qiagen) with minor modification. IBriefly, to 250 µL fecal sample, 180 µL Buffer ATL was added and mixed carefully. To this mixture, 59 µL of enzyme cocktail containing 50 µL lysozyme (10 mg/mL, Sigma-Aldrich), 6 µL mutanolysin (25 KU/mL, Sigma-Aldrich), and 3 µL lysostaphin (4,000 U/mL, Sigma-Aldrich) was mixed and the resuspended sample was transferred in to tubes containing 0.4 g sterile zirconia beads. The samples were homogenized in a Mini-BeadBeater at maximum speed for 1 minute and then incubated at 37°C for 30 minutes. A total of 20 µL of proteinase K was added and thoroughly mixed followed by incubation at 56°C for 1–2 hours to allow complete lysis. The samples were then centrifuged at maximum speed for 5 minutes to pellet debri. The supernatant was then used for DNA extraction using the DNeasy blood and tissue kit according to manufacturer's guidelines.

16S rRNA gene sequencing methods were adapted from the methods developed for the NIH-Human Microbiome Project (5). Filtered sequences with >97% identity were clustered into operational taxonomic units (OTU) and classified at the genus against the SILVA 16S ribosomal RNA sequence database (release 138.1; ref. 6). The relative abundance of each OTU was determined for all samples. A step-by-step description of our analysis pipeline has previously been published and is available (7). QIIME 2 platform was utilized for processing and visualizing microbiome data (8). All processed data are available as Supplementary Index S1.

### GM Analysis

Complete response (CR) was defined as pT0N0M0 pathologic assessment, partial response (PR) as (pTis, Ta, T1N0M0) and all other stages defined as CR. Response data were combined for CR and PR into single category to denote chemotherapy response with downstaging of the disease. Differentially abundant OTUs were identified using LEfSe [linear discriminant analysis (LDA) effect size] for each pairwise comparison of clinical groups [healthy vs. bladder cancer, bladder cancer vs. partner, CR vs. no response (NR)].

LEfSe first uses a nonparametric factorial Kruskal–Wallis rank-sum test to identify differentially abundant OTUs (9). This is followed by a set of pairwise tests among clinical groups to ensure biologic consistency using the Wilcoxon rank-sum test. LDA is then used to estimate the effect size of each differentially abundant OTU. LEfSe statistics were ranked to identify the greatest differences in microbial relative abundance across patient groups mentioned above. Associations between individual taxa and clinical variables were examined using generalized linear models as implemented in the Microbiome Multivariable with Linear Models (MaAsLin2) package (10).

To create a genus-level OTU table, OTUs with the same genus name were merged into one genus. We calculated the relative abundance of each genus. Samples within patients with bladder cancer were grouped into two clusters based on beta diversity using k-means clustering (11). The number of clusters (*k* = 2) was determined heuristically.

### Short Chain Fatty Acid Analysis

Rectal swab samples with ample stool and peripheral blood collected in Ethylenediaminetetraacetic acid (EDTA) tubes prior to cystectomy and administration of antibiotics was analyzed for short chain fatty acid (SCFA) composition in blood and stool was performed by Microbiome Insights. Briefly, SCFA extraction was replicated on the basis of that of Zhao and colleagues (12). Material were resuspended in MilliQ-grade H_2_O, and homogenized using MP Bio FastPrep, for 1 minute at 4.0 m/s. A total of 5 mol/L HCl was added to acidify fecal suspensions to a final pH of 2.0. Acidified fecal suspensions were incubated, and centrifuged at 10,000 RPM to separate the supernatant. Fecal supernatants were spiked with 2-Ethylbutyric acid for a final concentration of 1 mmol/L.

Extracted SCFA supernatants were stored in 2-mL GC vials, with glass inserts. SCFA were detected using gas chromatography (Thermo Fisher Scientific Trace 1310), coupled to a flame ionization detector (Thermo Fisher Scientific). Our SCFA column is “Thermo TG-WAXMS A GC Column, 30 m, 0.32 mm, 0.25 um,” which is very similar to the instrument and method we have taken from the literature.

Additional SCFA methodology can be found in Supplementary Methodology.

### Statistical Assessment

Alpha diversity comparison between bladder cancer versus healthy, CR versus NR, bladder cancer versus partner and enterotype were compared using Wilcoxon rank-sum or Mann–Whitney test (used for comparison between binary variables) and Spearman rank was used to compare continuous variables. Fisher exact test was used when proportions were compared between binary variables. Adjustments for multiple comparisons were done using the FDR method at an α level of 0.05. Hypothesis testing was done using both one-sided and two-sided tests as appropriate at a 95% significance level. Correlation analysis to investigate the relationship between SCFA and bacterial taxa and clinical response was assessed via Spearman correlation coefficient. All analyses were conducted in R (13) and Python (14). All visualization was performed via R, Python, with CorelDraw.

Random forest prediction for response-prediction classifier was utilized to test prediction performance of identified microbiome genera (15). AUC was used to assess the performance of the classifier for CR.

### Data Availability

Raw data can be provided upon request from the corresponding author. Analyzed data can be found in Supplementary Appendix.

## Results

### Microbiota Perturbations and Bladder Cancer–specific Alterations Compared with Healthy Controls

**FIGURE 1:**
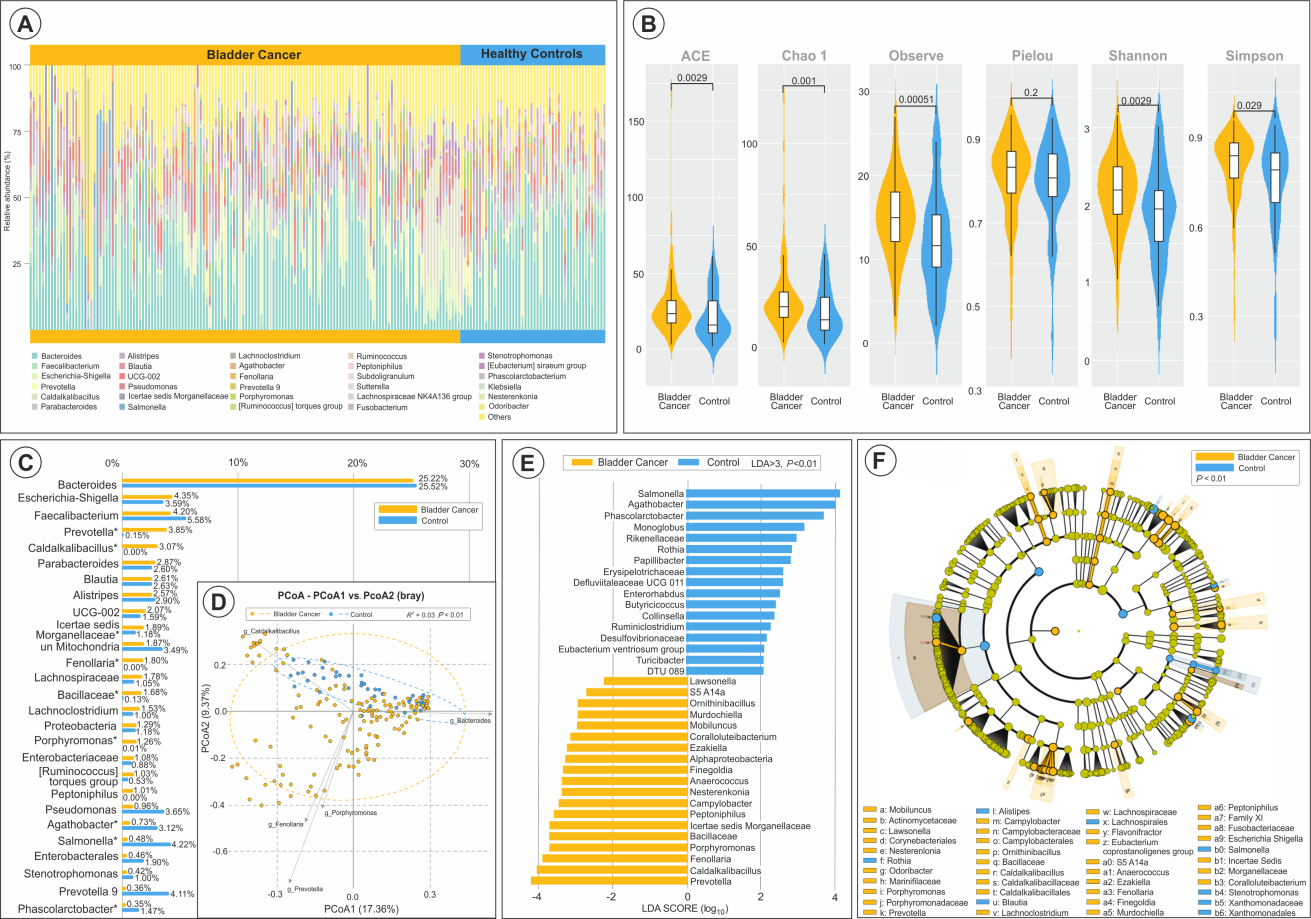
Bladder cancer versus controls microbiome diversity and differences. **A,** Stacked bar plot displaying the relative abundances of predominant bacterial genera identified in patients with bladder cancer (*n* = 142) and control individuals (*n* = 48). **B,** Distribution of alpha diversity indices across both cohorts, quantified using ACE, Chao1, Observed species, Pielou's evenness, Shannon, and Simpson indices, with error bars denoting variability within each group. **C,** Overall relative abundances of bacterial general identified by group (bladder cancer vs. control). **D,** Beta diversity assessed through weighted UniFrac PCoA, highlighting distinct microbial community structures associated with each group. **E,** LEfSe identifying differentially abundant taxa at various taxonomic levels, supported by a taxonomic cladogram visualized in **F**.

We first analyzed the GM differences between all available patients with bladder cancer (*n* = 142) and controls (CTR; *n* = 48). We observed a higher prevalence of *Prevotella* (4.35% vs. 0.17%, *P* = 0.001) and *Porphyromonas* (1.37% vs. 0.02%, *P* = 0.001) in patients with bladder cancer, along with a reduced abundance of *Faecalibacterium* (4.66% vs. 6.19%, *P* = 0.04) compared with controls. In addition, there was an overall increase in alpha diversity indices in the bladder cancer group (Shannon *P* = 0.00029, Simpson *P* = 0.0029, Chao *P* = 0.001, Observe *P* = 0.00051; Supplementary Index S1), although the differences were minor (Fig. 1A–C).

Principal coordinates analysis (PCoA) for beta diversity (Fig. 1D) demonstrated distinct microbial community separation between the groups, associated with disease presence (bladder cancer vs. CTR; Fig. 1D).

Using these data, we also identified differential bacterial abundance between the GM of healthy controls and patients with bladder cancer, reaffirming the previously noted increased levels of *Prevotella* and *Porphyromonas* in the bladder cancer group (LDA > 3.5, *P* < 0.01 for both; Fig. 1E and F).

In addition, we explored the influence of factors like gender on microbial composition, comparing the abundances of predominant bacteria such as *Bacteroides* (32.01% vs. 27.8%), *Prevotella* (4.2% vs. 5.5%), and *Escherichia coli* (4.0% vs. 6.9%) between males and females, respectively. Our analysis showed no statistically significant differences in these bacterial abundances between genders (*P* > 0.05; Supplementary Fig. S4A).

Furthermore, we examined alpha diversity, measured by the Shannon index, and found no significant differences between males and females (*P* = 0.47). This was further supported by the lack of discernible clustering between the two groups, as visualized in Supplementary Fig. S4B, S4C, and S4E). While there was a total of 64 unique OTUs identified in males, the majority of these were overlapping with those found in females (157 OTUs shared; Supplementary Fig. S4D). Our results indicate that, at least in the context of the abundances of the selected bacteria, alpha diversity, and clustering patterns, there were no notable differences observed between males and females.

### Demographic Covariates and Impact on Microbiome

**FIGURE 2:**
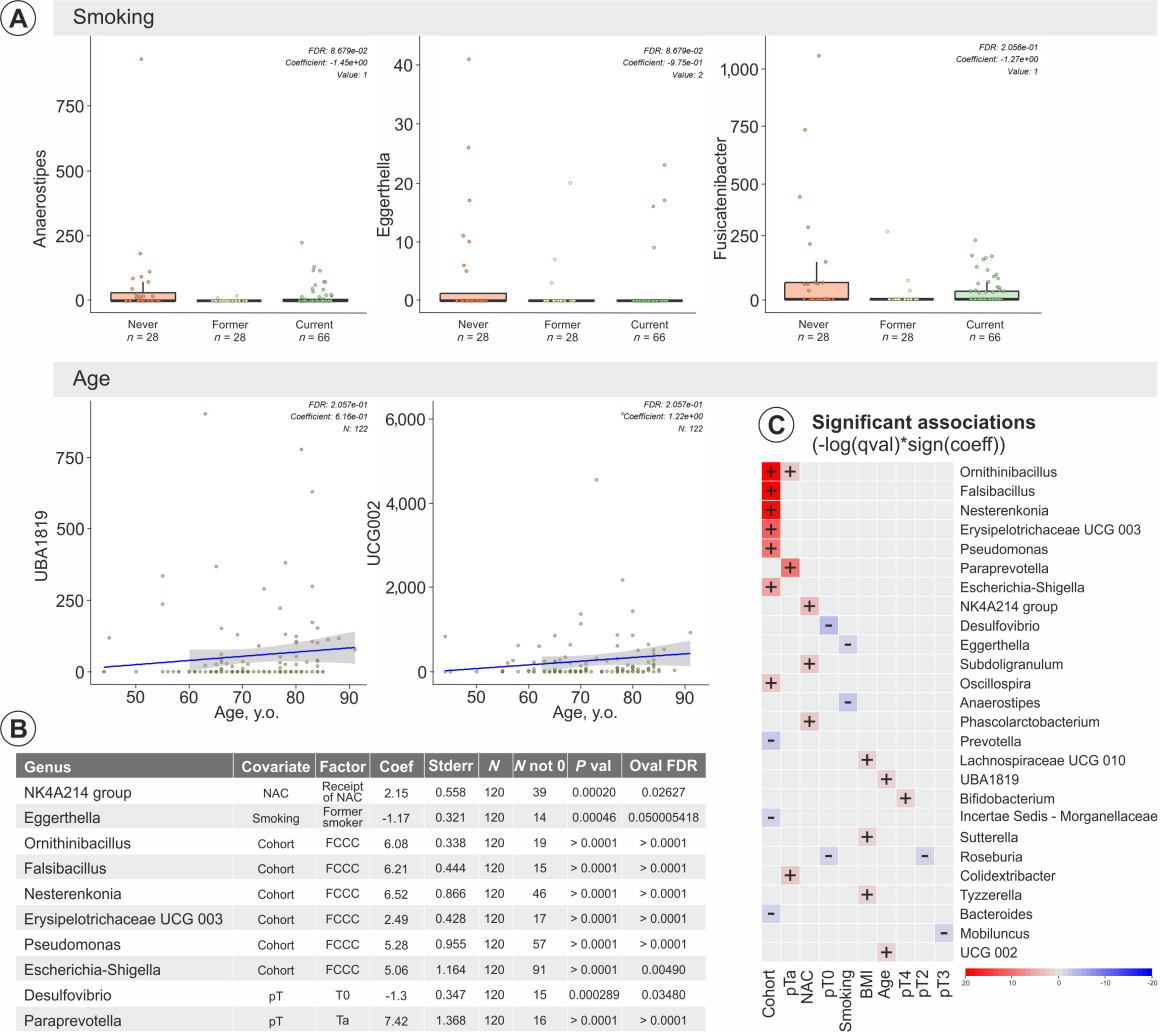
Correlations between participant demographics, clinical characteristics, and microbial taxa. **A,** Plots illustrating the impact of smoking status and age on specific microbial taxa, annotated with statistical significance and effect sizes. **B,** Comprehensive summary of significant correlations (FDR < 0.05) between participant characteristics—including smoking, age, BMI, treatment specifics, and institutional data—and microbial composition. **C,** Heat map representation of the strength and direction of correlations between participants’ characteristics and individual OTUs, underscoring the microbial shifts correlated with demographic and clinical variables.

We aimed to determine which demographic or outcome factors had the strongest associations with GM profiles by utilizing the MaAsLin2 analysis. This thorough analysis identified sex, smoking status, and cohort as significant covariates that were statistically associated with variations in bacterial profiles (Fig. 2A–C, Supplementary Appendix).

Specifically, smoking status had a noticeable effect on the bacterial abundance within the *Anaerostipes, Eggerthella*, and *Fusicatenibacte*r taxa (Fig. 2A). The differences in bacterial abundance were especially marked when comparing current smokers with former smokers. In addition, age was linked to changes in the *UBA1819* and *UCG0002* taxa, with older adults showing a higher association and prevalence of these bacterial profiles (Fig. 2B and C). The stage at the time of cystectomy and the institution were also associated with distinct microbiome profiles, notably affecting *Pseudomonas* and *Escherichia-Shigella* populations. Interestingly, a decreased abundance of *Desulfovibrio* was associated with a pT0 (no evidence of disease) status at cystectomy, even after adjusting for NAC, age, body mass index (BMI), sex, and variant histology.

### Bacteria Taxa Impact on NAC Treatment Response

**FIGURE 3:**
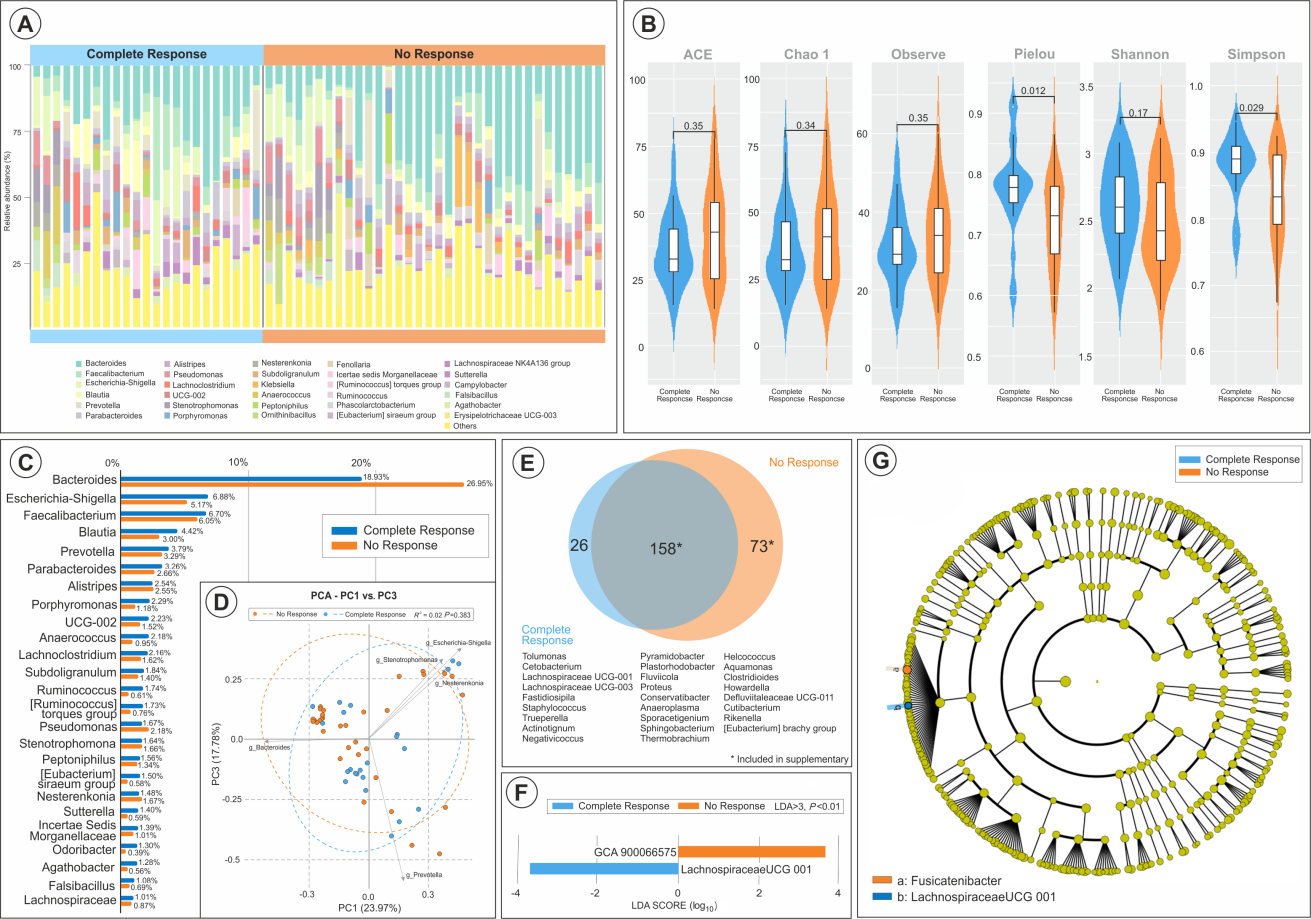
Microbial composition variance in relation to chemotherapy response in patients with bladder cancer. **A,** Proportional representation of the GM's phylogenetic composition in patients showing CR versus NR to NAC (Also depicted in **C**). **B,** Alpha diversity metrics compared across response groups, highlighting minimal statistical disparities. **D,** Weighted UniFrac PCoA depicting the lack of distinct microbial clustering by response category. **E**–**G,** Venn diagram and LEfSe analysis illustrating unique and differentially abundant microbial taxa associated with NAC responses, enriched in either CR or NR groups, along with a taxonomic cladogram delineating these associations.

Given the potential influence of compositional differences on both cancer development and therapeutic response, we investigated how specific GM compositions affect the response to NAC, as indicated by CR versus NR at the time of surgery. We analyzed GM OTU abundance in CR versus NR cases (*n* = 57), comparing the enrichment of OTUs in CR (*n* = 23) versus NR (*n* = 34) cases (Fig. 3A). No significant differences were observed in alpha diversity (Ace *P* = 0.35, Chao *P* = 0.34, Observe *P* = 0.35, Shannon *P* = 0.17) or beta diversity (*P* = 0.383) between the two groups (Fig. 3B and D). CR cases exhibited both overlapping and unique OTUs compared with NR, including taxa such as *Proteus, Fastidiosipila,* and *Tolumonas* (Fig. 3E). In contrast, NR cases showed a higher abundance of *Bacteroides* (26.95% vs. 18.93%, *P* < 0.01) and *Pseudomonas* (2.18% vs. 1.67%, *P* = 0.03) compared with CR (Fig. 3A and C). High-dimensional class comparisons using LEfSe, adjusted for overall abundance, identified *genus GCA-900066575* (an unidentified member of the Lachnospiraceae) as the only bacteria differentiating CR from NR, being enriched in NR subjects (LDA > 3, *P* < 0.01; Fig. 3F). In contrast, an increased abundance of *Lachnospiraceae*, known for producing SCFAs, was associated with the responder status (LDA > 3, *P* < 0.01; Fig. 3F and G).

### Community Abundances

Because of significant differences in composition and associations with responses to NAC regimens, we investigated whether collective abundances within specific treatments correlated with CR, beyond individual bacterial genera. We grouped all identified OTUs using hierarchical clustering without incorporating response data. This analysis revealed that the bladder cancer cohort divided into two groups, with the majority of patients with bladder cancer falling into community Group I (*n* = 115, 80.9%), which included many patients with a history of NAC (*n* = 48, 84.2%; Supplementary Fig. S5A). As expected, significant differences in alpha and beta diversity between the groups emerged because of the nature of the analysis (Supplementary Fig. S5B and S5C; Supplementary Appendix). Despite Group I containing a larger proportion of nonresponders compared with Group II (60.4% vs. 55.6%, *P* = 0.78), this difference was not statistically significant (Supplementary Fig. S5E and S5F).

Compared with Group II, Group I patients showed a higher abundance of *Bacteroides* (34.05% vs. 8.59%, *P* < 0.001) and a decrease in *Prevotella* (1.37% vs. 17.47%; *P* < 0.01; Supplementary Fig. S5A). Group I contained 136 unique OTUs, whereas Group II contained 19 unique OTUs (NR group; Supplementary Fig. S5D). Overall, the grouped communities within patients with bladder cancer did not predict NAC response status based on hierarchical clustering.

### GM Changes During Chemotherapy

Because the CW cohort samples were collected at the time of surgery post-NAC, our study then shifted focus to identify gut compositional differences in patients with bladder cancer before any treatment, during, and after chemotherapy, compared with their partners. To account for environmental influences such as diet, housing, and exposure, we also collected stool samples from live-in partners at the same timepoints. Throughout the course of chemotherapy, no significant changes were observed in alpha and beta diversity in patients with bladder cancer (Supplementary Fig. S6A, S6B, and S6F). However, there were noticeable fluctuations in composition, particularly an increase in the prevalence of Bacteroides, Escherichia-Shigella, and Roseburia after NAC compared with before (Supplementary Fig. S6A and S6D). Although eight OTUs were unique to the post-NAC samples (Supplementary Fig. S6E), the overall diversity and distribution remained similar across all three timepoints, with 102 OTUs common to each (Supplementary Fig. S6A–S6D and S6F). LDA showed no significant differences in GM composition before, during, or after chemotherapy (Supplementary Fig. S6C).

While both patients and partners showed no significant changes in diversity throughout the NAC period (Supplementary Fig. S6A–S6D, Supplementary Fig. S7B and S7C), patients with bladder cancer experienced increases in *Bacteroides*, *Escherichia-Shigella*, and *Roseburia* post-NAC, in contrast to their partners, whose abundances remained stable (Supplementary Fig. S7A and S7D–S7F).

### Stool and Serum SCFA Analysis

**FIGURE 4:**
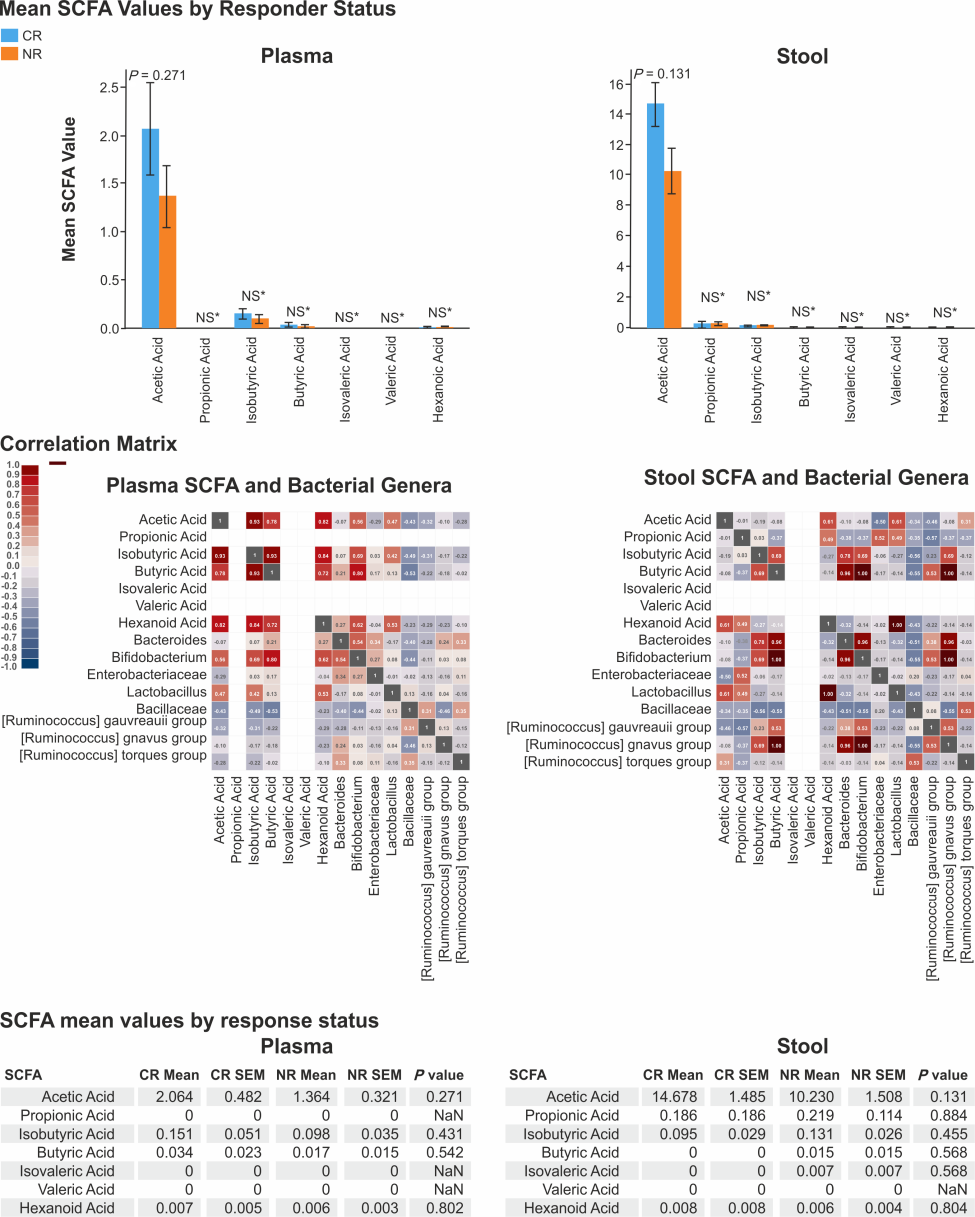
Analysis of SCFA concentrations in relation to chemotherapy response. Top: Mean SCFA concentrations in plasma and stool of patients with bladder cancer categorized by NAC response status (CR vs. NR), showing no significant differences. Bottom: Correlation matrices between SCFA levels and bacterial genera in plasma and stool, with the strongest positive and negative correlations marked, indicating the metabolic interactions within the GM in the context of cancer therapy response.

In addition to assessing GM differences in our cohorts, we expanded our analysis to include SCFA concentration differences in stool and plasma among the cohorts. We specifically measured the levels of acetic, propionic, isobutyric, butyric, isovaleric, valeric, and hexanoic acids in both the responder and nonresponder groups (*n* = 18), as summarized in Fig. 4.

Our results showed no statistically significant differences in SCFA concentrations between the two groups (CR vs. NR) in either stool or plasma (Fig. 4). However, we noted a significant difference in the average concentration of acetic acid, which was substantially higher in fecal samples than in plasma (14.67 ± 1.48 vs. 2.06 ± 0.48, *P* < 0.001). While all tested SCFAs were detectable in stool, only propionic, isovaleric, and valeric acids showed minimal presence in plasma.

In addition, when examining the impact of NAC on plasma SCFA levels, we observed an increase in detectable levels of acetic acid (1.56 vs. 0.71, *P* = 0.12), isobutyric acid (0.036 vs. 0.01, *P* = 0.01), and butyric acid (0.016 vs. 0.01, *P* = 0.01) in patients who underwent NAC compared with those who did not (Supplementary Appendix). Further analysis of the association between SCFA-producing bacteria (including *Bacteroides, Bifidobacterium, Enterobacteriaceae, Lactobacillus, Bacillaceae,* and *Ruminococcus*) and SCFA levels revealed no positive correlations in stool or blood (Supplementary Appendix). However, broader analysis identified a correlation between increased fecal isobutyric acid levels and higher abundances of *Akkermansia* (rs = 0.51, *P* = 0.017) and *Clostridia* (rs = 0.52, *P* = 0.018). In addition, *Lactobacillus* (rs = 0.49, *P* = 0.02) and *Enterobacteriaceae* (rs = 0.52, *P* < 0.03) showed positive correlations with elevated propionic acid levels (Supplementary Appendix).

Finally, our univariate analysis exploring the associations between SCFA concentrations in blood and stool and NAC response status did not yield any statistically significant findings (Supplementary Appendix).

### Microbiome Response-Predictor Classifier

**FIGURE 5:**
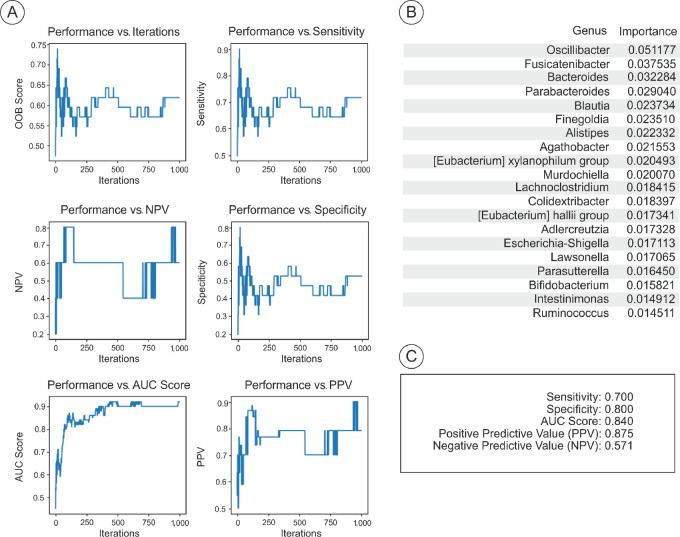
Predictive efficacy of microbial biomarkers on NAC response. **A,** Graphical representation of model performance metrics (out of bag score, sensitivity, NPV, specificity, PPV, AUC score) across iterations, evaluating the robustness of microbial biomarkers in predicting NAC response. **B,** Ranked importance of bacterial genera as predictors, derived from a machine learning model assessing their utility in distinguishing between CR and NR. **C,** Summary statistics including sensitivity, specificity, PPV, NPV, and overall AUC score, affirming the predictive value of microbial signatures in clinical outcomes. PPV, positive predictive value; NPV, negative predictive value.

We utilized a random forest machine learning approach to develop a phenotype prediction classifier for CR in patients with MIBC, based on a set of microbial genera. Our analysis included 57 stool samples. After conducting five rounds of 1,000-fold cross-validation, microbial variables emerged as the optimal predictors for CR, achieving an AUC of 0.88 (95% confidence interval: 0.81–0.94; Fig. 5A) with a sensitivity of 0.70, specificity of 0.80, positive predictive value of 0.88, and a negative predictive value of 0.571 (Fig. 5C). In the machine learning model, the genera *Oscillibacter, Fusicatenibacter*, and *Bacteroides* were identified as having the highest levels of importance (0.051, 0.037, and 0.032 respectively), although no single genus exhibited a dominance, indicating a complex multigenera influence in CR (Fig. 5B).

In addition, we assessed 24 clinical variables to evaluate their potential in predicting CR. Following the same modeling process, the AUC for these clinical factors was 0.50, indicating no discriminatory power based solely on clinical and pathologic factors for predicting CR, unlike the microbiome signature (Supplementary Appendix). This suggests that predictions for NAC response are feasible based on a patient's microbiome profile, rather than clinical characteristics alone.

### Microbiome Composition During BBN-induced Tumorigenesis

**FIGURE 6:**
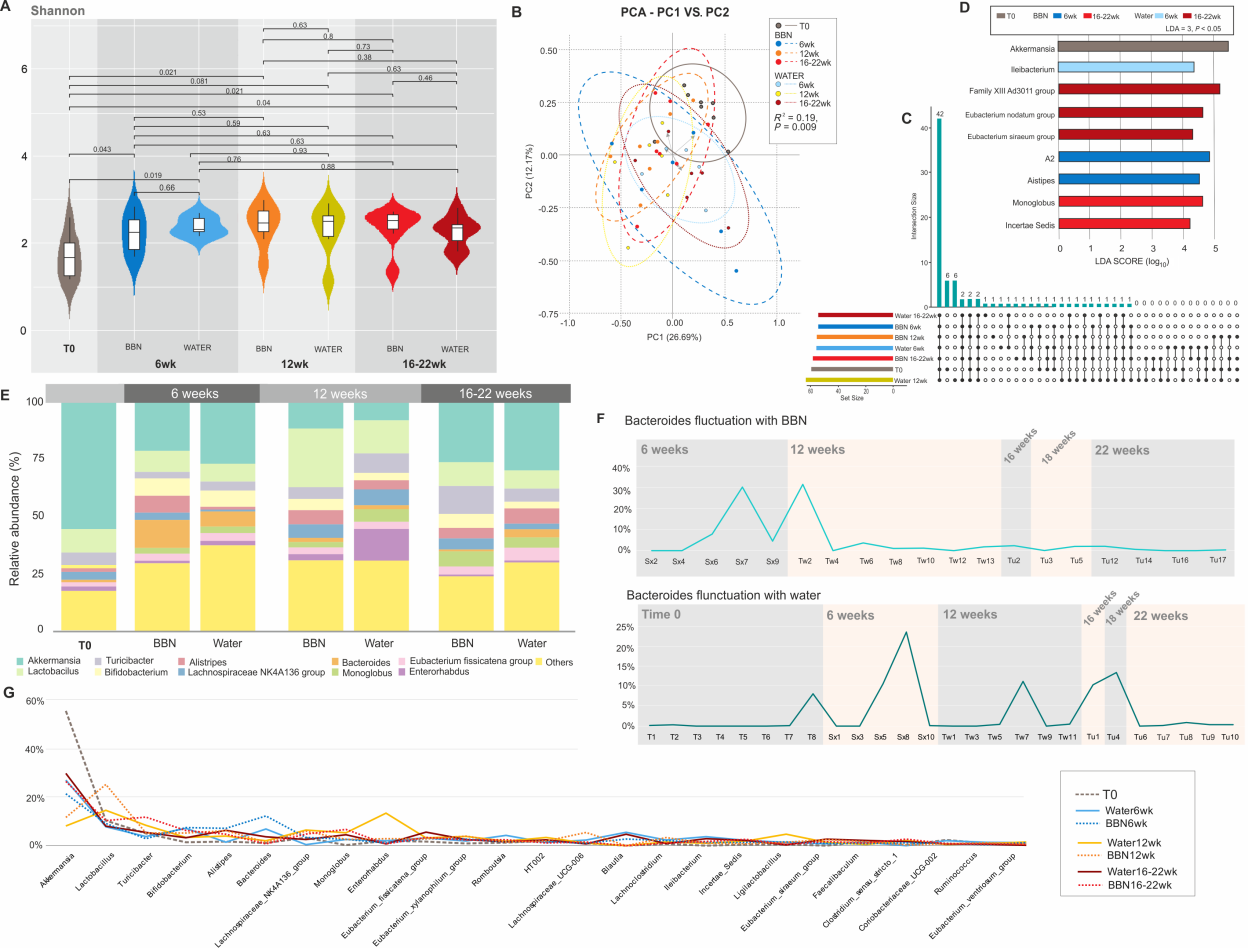
Longitudinal microbiota analysis in a murine model exposed to BBN versus water. **A,** Shannon diversity index measures across different timepoints (0, 6, 12, 16–22 weeks) showing changes in alpha diversity in mice treated with BBN compared with those given water. Each point represents a time-specific diversity score, providing insights into the temporal dynamics of microbial diversity. **B,** PCoA plot of beta diversity based on Weighted UniFrac distances, illustrating the microbial community variations over time between BBN-treated and control groups. Significant differences are highlighted, demonstrating the impact of BBN exposure on microbial community structure. **C,** LDA scores quantifying the effect sizes of differentially abundant taxa between the BBN and water groups at each assessed timepoint. This visualization identifies specific taxa that are significantly impacted by BBN treatment. **D,** Bar graphs of the relative abundances of key bacterial taxa at different timepoints for BBN-treated versus water-treated mice. This detailed breakdown provides a clearer understanding of how specific bacterial populations fluctuate in response to carcinogen exposure. **E,** Relative abundance profiles of major bacterial genera across all timepoints, comparing BBN and water treatments. **F,** Fluctuation patterns of Bacteroides prevalence in the BBN and water groups, plotted across the study duration. **G,** Temporal trajectories of various bacterial OTUs between BBN and water groups, providing a longitudinal view of microbial changes and stability in response to treatment.

To assess GM changes associated with tumorigenesis, stool samples from mice given BBN (a bladder carcinogen) or regular drinking water were collected and analyzed at four timepoints: (i) baseline, (ii) during treatment, (iii) at the end of BBN exposure, and (iv) when tumors were visible radiographically, just before sacrifice. This was done to account for age-related changes. Our initial analysis of the longitudinal changes within the control and BBN-exposed mice showed no differences between the groups at individual timepoints (Fig. 6C, E, and G). While alpha diversity was not significant (Fig. 6A), beta diversity analysis did reveal clustering at time zero compared with later timepoints, suggesting changes were due to gut maturation rather than exposure to BBN (Fig. 6B).

Given the observed differences in Bacteroides abundance in patients with bladder cancer at different institutions, we monitored the abundance of *Bacteroides* in both BBN-exposed and control mice throughout the experiment. We observed a higher prevalence of *Bacteroides* in BBN-exposed mice (12.30% vs. 6.89%; Supplementary Index S1; Supplementary Fig. S8F–S8J), which interestingly normalized to control levels by the end of the 12-week BBN exposure period (Supplementary Fig. S8G). In both BBN and water control groups, there was a higher abundance of *Akkermensia* and *Verrucomicrobiota* at pretreatment, which then declined at subsequent timepoints (6, 12, 16–22 weeks), regardless of exposure (LDA 5, *P* < 0.05; Supplementary Fig. S8B–S8E and S8G–S8J). There were no differences in alpha diversity by exposure and week (Supplementary Fig. S8A and S8B), although week-specific clustering was present among both groups (Supplementary Fig. S8D and S8I).

Considering the potential for sex-dependent changes in microbial composition (16), we also analyzed compositional differences in the microbiome in response to BBN exposure by sex. No significant differences in alpha and beta diversity (Supplementary Fig. S9A–S9D, S9F–S9I) were observed in males or females separately throughout the experiment. Week-dependent differences in overall abundances were seen in both males and females (Supplementary Fig. S9B and S9G), although not significant with further LDA analysis (Supplementary Fig. S9E and S9J).

## Discussion

In this article, we report the most extensive study to date on the association between the GM and the response to NAC in MIBC. We not only explored differences between control (CTR) and patients with bladder cancer but also examined changes in the GM throughout NAC. A significant observation from our study is the link between the presence of Bacteroides and poor NAC response in patients with MIBC. This association is not limited to bladder cancer; similar reductions in the effectiveness of both immunotherapy and chemotherapy have been noted in cases of melanoma (7), pancreatic (17), and colon cancers (18). Our findings align with previous research suggesting a connection between GM and tumorigenesis, particularly noting increases in Prevotella and Bacteroides in patients with bladder cancer, which are commonly associated with both tumorigenesis and therapy response (19–21).

Our data indicate that both the baseline gut microbiota and its changes during chemotherapy are crucial predictors of CR. The microbial signatures we identified showed better predictive power for CR outcomes post-NAC than traditional clinical variables alone. However, for improved accuracy and reliability, future studies should consider integrating microbial analyses with clinical data, genomic classifiers, and circulating tumor DNA. The primary goal of our research is to identify patients unlikely to benefit from NAC, who might instead benefit from alternative systemic treatments or early surgery. By initiating the characterization of a predictive microbial signature, we encourage further large-scale studies to validate our results and promote clinical trials integrating GM modulation for therapeutic advantages, along with a combination of tumor, host, and microbial variables.

The dynamics of GM changes during NAC in bladder cancer have not yet been thoroughly examined. However, existing studies in colorectal (22) head and neck (23) and cervical (24) cancers have all documented significant microbiome fluctuations throughout treatment courses. In line with these findings, our study observed that the longitudinal changes in the microbiota were primarily marked by an increase in Bacteroides during NAC. A unique aspect of our research is the inclusion of patients’ live-in partners as a control to mitigate environmental influences on microbiome changes. Notably, while patients showed an increase in Bacteroides at the end of treatment, their partners did not exhibit similar shifts, supporting the notion of a genuine microbiota alteration rather than an incidental change. In addition, previous studies have highlighted a correlation between close social relationships and the composition of gut and skin microbiota, with the greatest similarities found between spouses (25). Our findings corroborate this, suggesting that the microbiome composition of couples exhibits notable concordance.

It is important to also highlight an interesting yet still controversial notion of existence of “enterotypes” characterized by dominant genera (*Bacteroides, Prevotella, and Ruminococcus),* and their co-occurring dietary traits, first published by Arumugam and colleagues (26) In particular, the *Bacteroides-*dominant enterotype was found to be associated with high animal protein and saturated fat, while the *Prevotella* enterotype was observed with high fiber/plant-based nutrition (low meat). As there is growing awareness of importance of dietary phenotype with health and disease and recognition of host-microbiome mutualism, it is difficult to say whether the dietary phenotype in our study (high fiber/plant-based *Prevotella* subtype) and response to NAC is result of a modifiable intervention or confounding by an environmental factor.

Our analysis of human plasma and fecal samples revealed no significant differences in concentration levels between the responder and nonresponder groups in assessing the efficacy of NAC. Although the variations in acetic acid levels in stool and plasma were not statistically significant, a consistent elevation was noted in patients who achieved a CR. This finding is in line with research on other types of cancer, such as urothelial carcinoma, highlighting acetic acid's role in promoting cellular apoptosis and its potential association with improved progression-free survival in colorectal cancer (27). Furthermore, the levels of acetic, propionic, isobutyric, butyric, isovaleric, valeric, and hexanoic acids in both fecal and plasma samples showed no correlation with known SCFA producers like *Bacteroides, Bifidobacterium, Enterobacteriaceae, Lactobacillus, Bacillaceae,* and *Ruminococcu*s, contrary to what is commonly reported in literature. Previous research has suggested that propionate might boost the antitumor effects of cisplatin in human hepatocellular carcinoma cells (28), yet we found no such correlation in patients with bladder cancer. Likewise, although butyrate is reported to increase apoptosis and suppress growth, migration, and invasion in gastric cancer cell lines treated with cisplatin, our study did not find a relationship between SCFA levels and NAC response (29).

Our study was designed to track the longitudinal changes in mice exposed to a carcinogen, aiming to pinpoint crucial moments of transformation and dysbiosis. In our experiments, mice exposed to BBN exhibited a consistent increase in *Bacteroides* up to week 12, the point at which BBN exposure was halted, followed by a rapid decrease once the carcinogen was withdrawn. While the gut microbiotas of humans and mice share considerable similarity, with overlaps of 90% at the phylum level and 89% at the genus level, differences at the species level are significant. For example, our study noted a steady increase in Bacteroides during the 12-week BBN exposure period. In mice, this genus is primarily represented by the S24-7 family, especially *Bacteroides ovatus*, whereas in humans, *Bacteroides vulgatus* is more common (30). While subtle differences in microbiome composition exist, employing murine models for microbiome-based therapeutic research is essential for developing new treatments in a controlled setting that is not possible in human studies. Although the mice in our study did not receive treatment for their BBN-induced tumors, limiting the implications of these findings, our research does provide evidence supporting the potential for Bacteroides manipulation in murine models, particularly for future therapeutic developments.

Our study has several limitations. While we collected clinical data for patients, we did not acquire concurrent tumor data such as genomic mutations and immune infiltration, which are critical for enhancing the response classifier. In addition, although we excluded patients who received antibiotics within 6 weeks prior to therapy, we did not assess nutritional data or over-the-counter medications that might influence the GM. However, our study is distinct in its inclusion of patients’ partners, which allowed us to adjust for environmental exposures that could affect microbiome composition. Moreover, because our patients received different NAC regimens—GC and MVAC—the differences in stool microbiome observed with MVAC may not apply to patients treated with GC, and vice versa. This necessitates further evaluation in a prospective setting among both GC and MVAC patient populations.

In conclusion, our findings indicate that certain microbial features in the baseline GM of patients with bladder cancer are linked to chemotherapy response. This is supported by real-world cohorts, highlighting an increased abundance of Bacteroides as a negative prognostic factor for CR. The data presented call for further validation in a large, prospective trial that also integrates immune and genomic markers from the host and tumor. We hope these preliminary results are promising and pave the way for additional ongoing trials.

## Supplementary Material

Supplementary Data Supplemental Data

Supplementary Table 1 Patient Characteristics

Supplementary Figure 1 Supplementary Figure 1: Overview of Sample Collection and Experimental Setup Diagram outlining the protocols for human fecal sample collection across various time points during chemotherapy in bladder cancer patients, accompanied by the setup of the murine bladder cancer model to study the gut microbiome.

Supplementary Figure 2 Supplementary Figure 2: Patient Recruitment Flow Chart Flowchart illustrating the step-by-step recruitment process of bladder cancer patients, detailing inclusion and exclusion criteria and subsequent enrollment stages

Supplementary Figure 3 Supplementary Figure 3: Imaging of Murine Urothelial Cancer CT urography and ultrasound images displaying bladder tumors induced by BBN in mice, shown in both axial and coronal views, with tumors highlighted by arrows.

Supplementary Figure 4 Supplementary Figure 4: Sex-Specific Microbiome Diversity in Bladder Cancer and Control Groups
A: Stacked bar plot showing the phylogenetic composition of bacterial taxa at the genus level in male and female cohorts of bladder cancer patients and controls.
B: Analysis of differentially abundant bacteria across taxonomic levels up to genus, with FDR adjustment.
C and D: Alpha and beta diversity metrics demonstrating microbial community segregation by sex and disease status through Weighted UniFrac PCoA.
E: Cladogram highlighting significant microbial differences across taxonomic levels with dot color coding by group.

Supplementary Figure 5 Supplementary Figure 5: Community Grouping Based on Gut Microbiome Response Status
A: Hierarchical clustering identifying two distinct microbial community groups among bladder cancer patients (CR and NR).
B and C: Alpha and beta diversity analyses illustrating significant differences and clustering patterns among the identified community groups.
D and E: Distribution of unique and shared bacterial OTUs across the two main groups, detailing their correlation with patient response status and individual group composition (F).

Supplementary Figure 6 Supplementary Figure 6: Gut Microbiome Dynamics During Neoadjuvant Chemotherapy
A: Relative abundance of major bacterial genera before, during, and after chemotherapy. Each color represents a different genus, with patient-specific changes highlighted to illustrate shifts in microbial composition over the course of treatment.
B: Violin plots representing alpha diversity indices (ACE, Chao1, Observed, Pielou, Shannon, and Simpson) at three stages of chemotherapy: before, during, and after treatment.
C: Cladogram derived from LEfSe analysis illustrating the most significantly altered taxa at each treatment stage. Colored nodes indicate taxa with statistically significant differences in abundance, with arrows highlighting key changes before, during, and after chemotherapy.
D: Each segment shows the percentage composition of each genus by treatment stage.
E: Venn diagram summarizing the overlap and unique bacterial taxa found at each stage of chemotherapy, quantifying shared and exclusive genera before, during, and after treatment.
F: Principal Coordinates Analysis (PCoA) plot based on weighted UniFrac distances, showing the clustering of microbial communities at different treatment stages.
G: Bar graph illustrating the proportional changes in specific bacterial genera over the course of chemotherapy, quantified and compared across three phases.

Supplementary Figure 7 Supplementary Figure 7: Gut Microbiome Changes in Partners of Bladder Cancer Patients
A: Stacked bar chart representing the relative abundances of key bacterial genera before, during, and after the chemotherapy periods in partners of patients.
B: Principal Coordinates Analysis (PCoA) plot based on weighted UniFrac distances showing the clustering of microbiome samples at different stages of chemotherapy (before, during, after). The ellipses represent the 95% confidence intervals for each group.
C: Violin plots illustrating the distribution of alpha diversity indices (ACE, Chao1, Observed, Pielou, Shannon, Simpson) at three key stages of chemotherapy.
D: Bar graph depicting the percentage representation of selected bacterial genera across the three phases of chemotherapy in partners.
E: Linear Discriminant Analysis (LDA) effect size (LEfSe) plot comparing bacterial taxa before and after chemotherapy, identifying which taxa are statistically significantly different between the two time points.
F: Venn diagram illustrating the unique and shared bacterial taxa found at each phase of chemotherapy.

Supplementary Figure 8 Supplementary Figure 8: Microbial Composition Differences in Mice Exposed to BBN Versus Water
Water Group:
A: Alpha diversity indices (ACE, Chao1, Shannon) plotted over multiple time points (0, 6, 12, 16-22 weeks), showing variations in microbial diversity in mice given water.
B: Line graph depicting the percentage composition of bacterial families over time.
C: Venn diagram highlighting the unique and shared bacterial operational taxonomic units (OTUs) at different time points.
D: Principal Coordinates Analysis (PCoA) based on weighted UniFrac distances.
E: Bar charts representing the relative abundance of specific taxa, identified as significantly different by LEfSe analysis, across the time points. The bars indicate changes in key microbial taxa that are statistically significant.
BBN Group:
F: Alpha diversity indices (ACE, Chao1, Shannon) for the BBN group.
G: Line graph showing the dynamic changes in the percentage composition of bacterial families over time in the BBN-treated mice.
H: Venn diagram detailing unique and shared bacterial OTUs between the different time points in the BBN group, illustrating the impact of the carcinogen on microbial turnover.
I: PCoA plot for the BBN group, using weighted UniFrac distances to show clustering patterns that differ from the water group.
J: LEfSe analysis bar charts showing the relative abundance of significantly different taxa across time points in the BBN group.

Supplementary Figure 9 Supplementary Figure 9: Microbial Composition Variability by Sex in BBN-Exposed Mice
BBN Gender Female:
A: Alpha diversity indices (ACE, Chao1, Shannon) across different time points (0, 6, 12, 16-22 weeks), showing variations in microbial diversity in female mice.
B: Stacked bar chart illustrating the relative abundance of bacterial genera at each time point.
C: Dot plot depicting the percentage of specific bacterial families at different time points, providing a quantitative view of how predominant families fluctuate with BBN exposure.
D: Principal Coordinates Analysis (PCoA) plot based on weighted UniFrac distances, showing the clustering of microbial communities across time, with color-coded points representing different time points to visualize the trajectory of community changes.
E: Bar chart with Linear Discriminant Analysis (LDA) scores of bacterial taxa that are significantly different across the studied time points.
BBN Gender Male:
F: Alpha diversity indices (ACE, Chao1, Shannon) displayed across the same time points as the female group, illustrating the microbial diversity dynamics in male mice.
G: Stacked bar chart showing the relative abundance of bacterial genera in male mice, and the specific microbial shifts unique to male physiology under BBN influence.
H: Dot plot of the percentage representation of bacterial families over time, helps in identifying sex-specific microbial behavior in response to carcinogenic treatment.
I: PCoA plot for the male group, illustrating shifts in microbial community structure with different clusters for each time point.
J: LDA score bar chart for male mice displaying significant microbial taxa changes over time providing insight on impact of BBN exposure on the male gut microbiome.

Supplementary Figure Legends Supplementary Figure Legends
