# Supplementary material for: Role of Gut Microbiome in Neoadjuvant Chemotherapy Response in Urothelial Carcinoma: A Multi-institutional Prospective Cohort Evaluation
Source: Cancer Res Commun. 2024 Jun 17;4(6):1505–16. doi: 10.1158/2767-9764.CRC-23-0479 (PMC11181990; doi:10.1158/2767-9764.CRC-23-0479)
Supplement: Supplementary Table 1 — Patient Characteristics [file crc-23-0479-s02.docx]

| Characteristic Table | | |
| --- | --- | --- |
| **Characteristic** | **Bladder Cancer (n=142)** | **Controls (n=48)** |
| **Gender** | N (%) | N (%) |
| Male | 112 (78.87) | 38/48 (79.17) |
| Female | 30 (21.13) | 10/48 (20.83) |
| **Age** |  |  |
| Median (interquartile range) | 74 (63-77) | 72 (62.75-77) |
| **BMI** |  |  |
| Median (interquartile range) | 28.7 (25.0-32.1) | 26.43 (23.89-30.47) |
| **Intravesical therapy** |  |  |
| Received | 4 (2.83) | N/A |
| Not Received | 137(96.47) |  |
| Unknown | 1(0.7) |  |
| **Final stage** |  |  |
| pT0 | 36 (25.35) |  |
| pTis | 17 (11.97) |  |
| pT2 | 56 (39.43) |  |
| pT3 | 33(23.23) |  |
| **NAC** |  |  |
| Yes | 57 (40.14) |  |
| No | 85 (59.86) |  |
| **Charlson Comorbidity Index (CCI)** |  |  |
| Median (interquartile range) | 3 (2-4) |  |
| Range | 2-6 |  |
| **Tumor Histology** |  |  |
| Urothelial | 130 (91.54) |  |
| Squamous | 6 (4.21) |  |
| Micropapillary | 4 (2.82) |  |
| Small cell | 2 (1.41) |  |
| **Nodes Positive** |  |  |
| Positive | 24 (16.91) |  |
| Negative | 118 (83.09) |  |
| **Smoking Status** |  |  |
| Current | 13 (9.15) | - |
| Former | 91 (64.08) | 10 (20.83) |
| Never | 27 (19.02) | 34 (70.83) |
| Unknown | 11 (7.75) | 4 (8.34) |

**Supplementary Table 1: Patient Characteristics**
