# Supplementary figures and images for: Role of Gut Microbiome in Neoadjuvant Chemotherapy Response in Urothelial Carcinoma: A Multi-institutional Prospective Cohort Evaluation
Source: Cancer Res Commun. 2024 Jun 17;4(6):1505–16. doi: 10.1158/2767-9764.CRC-23-0479 (PMC11181990; doi:10.1158/2767-9764.CRC-23-0479)

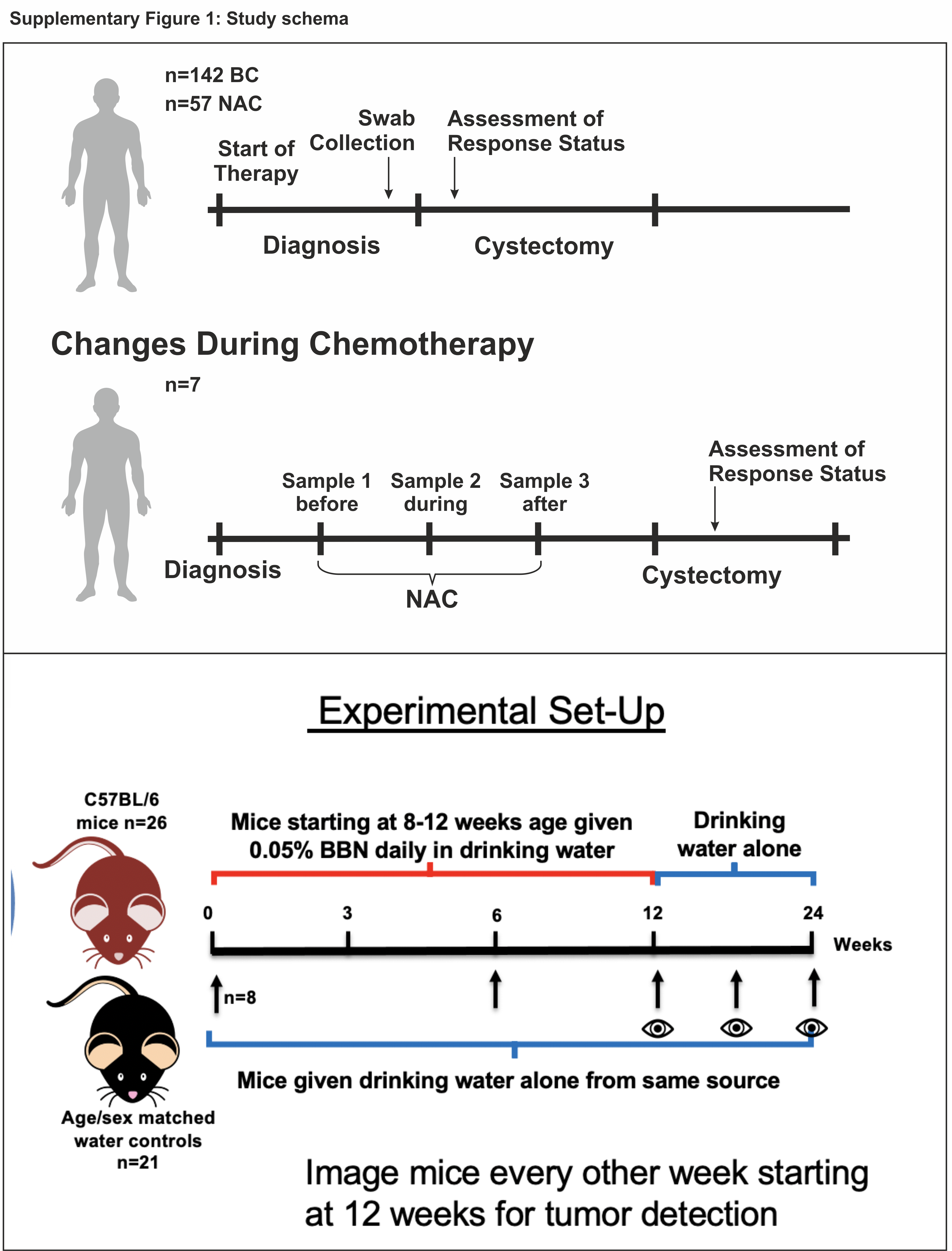

Supplement: Supplementary Figure 1 — Overview of Sample Collection and Experimental Setup Diagram outlining the protocols for human fecal sample collection across various time points during chemotherapy in bladder cancer patients, accompanied by the setup of the murine bladder cancer model to study the gut microbiome. [file crc-23-0479-s03.png]

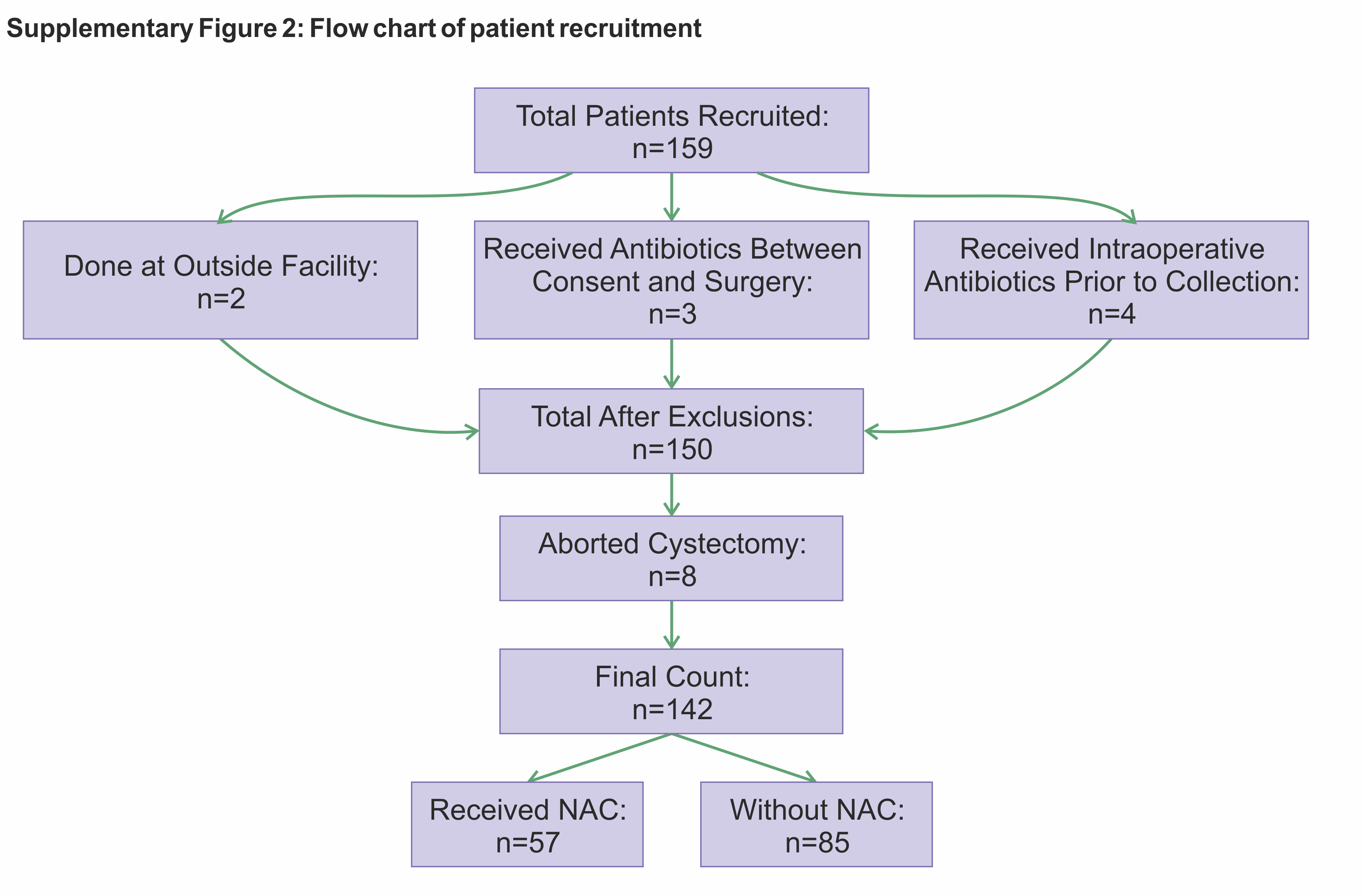

Supplement: Supplementary Figure 2 — Patient Recruitment Flow Chart Flowchart illustrating the step-by-step recruitment process of bladder cancer patients, detailing inclusion and exclusion criteria and subsequent enrollment stages [file crc-23-0479-s04.png]

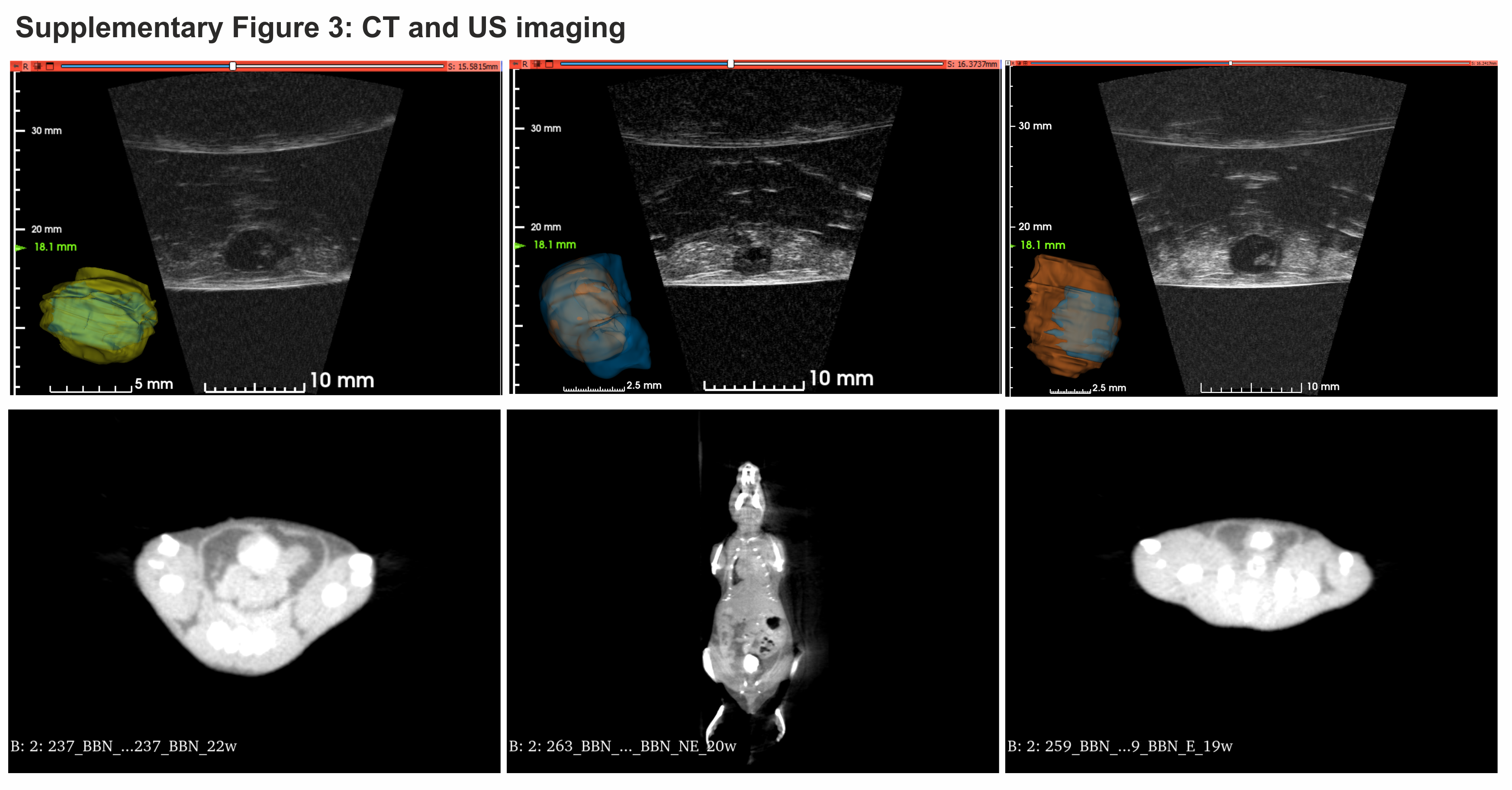

Supplement: Supplementary Figure 3 — Imaging of Murine Urothelial Cancer CT urography and ultrasound images displaying bladder tumors induced by BBN in mice, shown in both axial and coronal views, with tumors highlighted by arrows. [file crc-23-0479-s05.png]

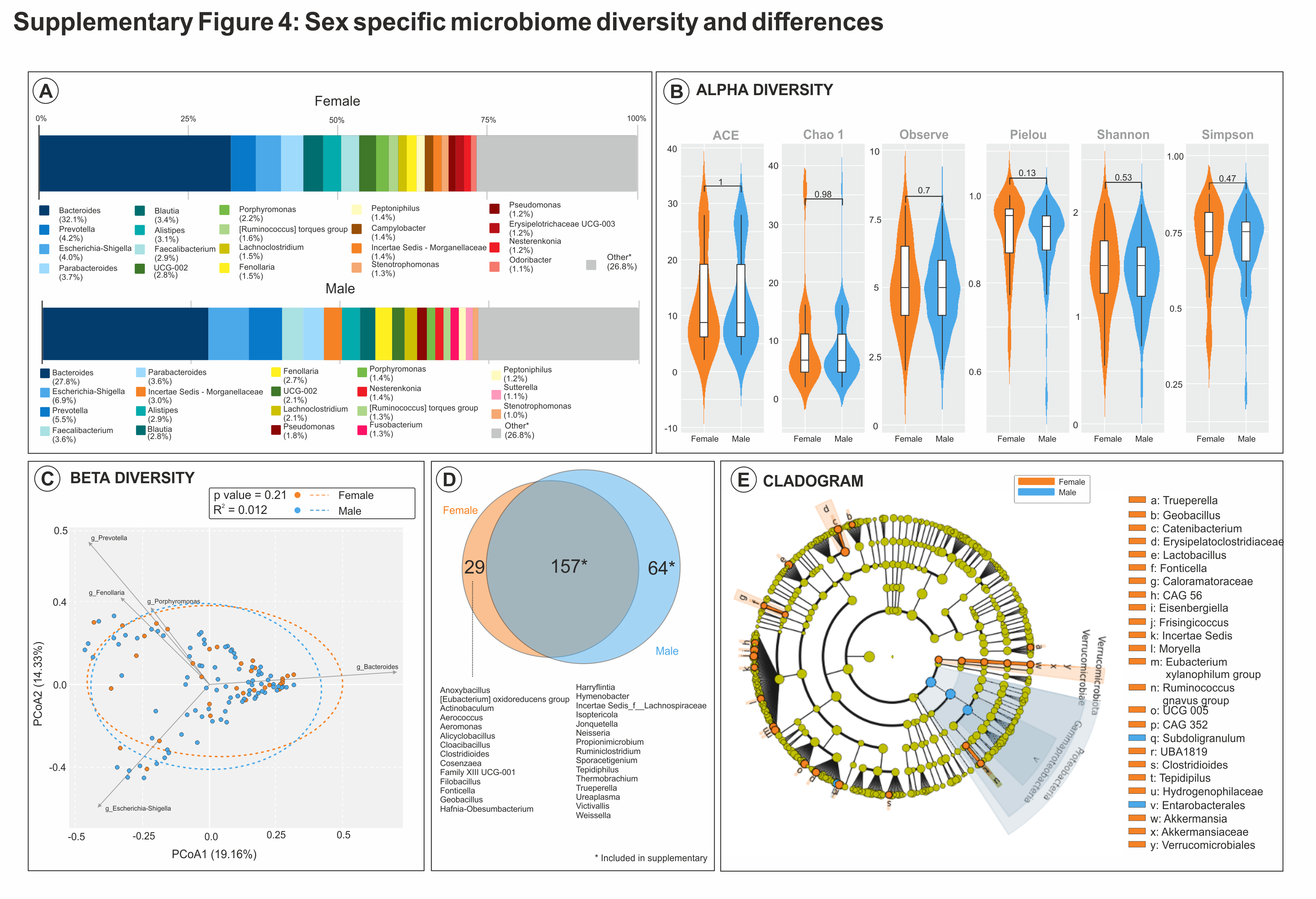

Supplement: Supplementary Figure 4 — Sex-Specific Microbiome Diversity in Bladder Cancer and Control Groups A: Stacked bar plot showing the phylogenetic composition of bacterial taxa at the genus level in male and female cohorts of bladder cancer patients and controls. B: Analysis of differentially abundant bacteria across taxonomic levels up to genus, with FDR adjustment. C and D: Alpha and beta diversity metrics demonstrating microbial community segregation by sex and disease status through Weighted UniFrac PCoA. E: Cladogram highlighting significant microbial differences across taxonomic levels with dot color coding by group. [file crc-23-0479-s06.png]

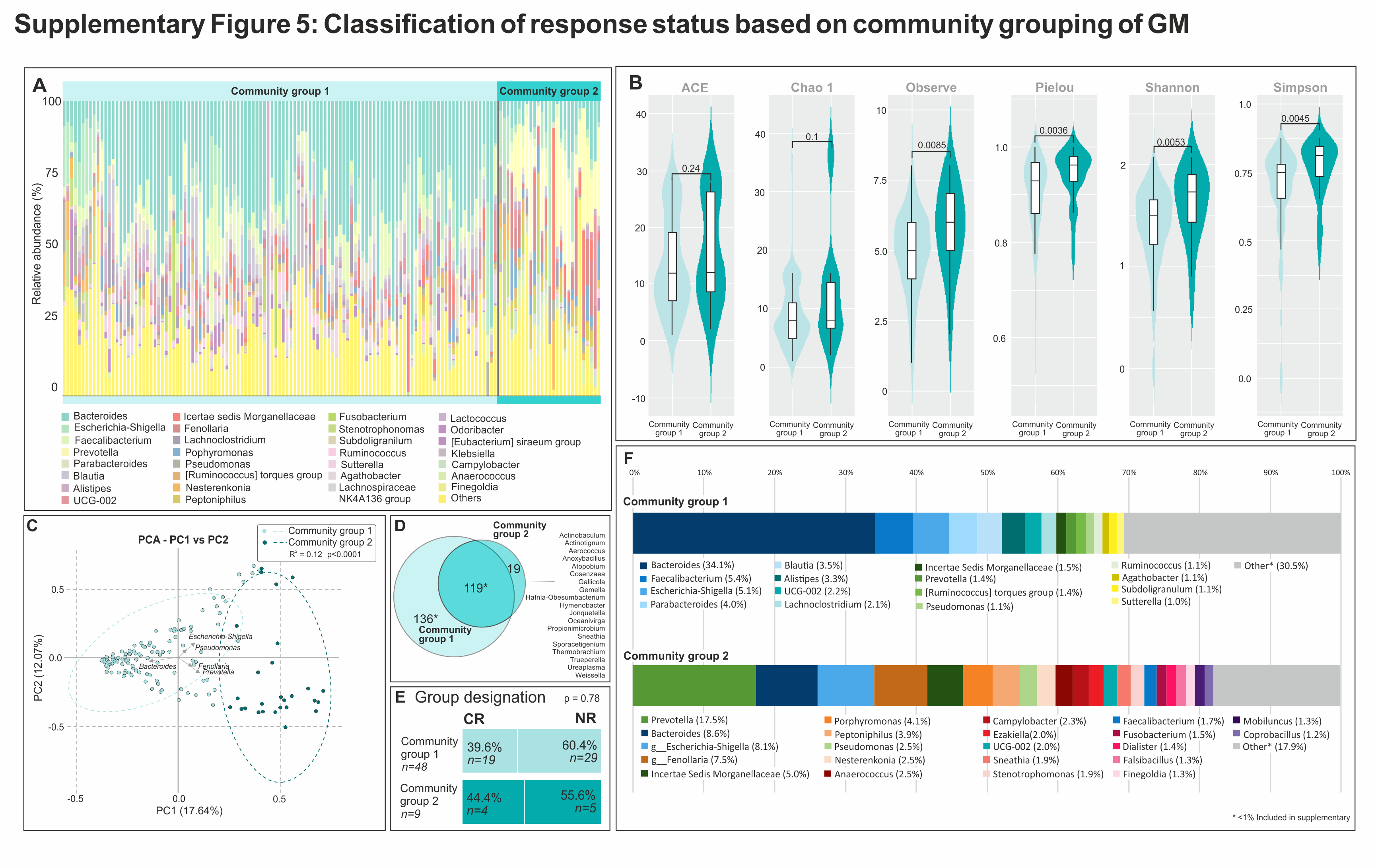

Supplement: Supplementary Figure 5 — Community Grouping Based on Gut Microbiome Response Status A: Hierarchical clustering identifying two distinct microbial community groups among bladder cancer patients (CR and NR). B and C: Alpha and beta diversity analyses illustrating significant differences and clustering patterns among the identified community groups. D and E: Distribution of unique and shared bacterial OTUs across the two main groups, detailing their correlation with patient response status and individual group composition (F). [file crc-23-0479-s07.png]

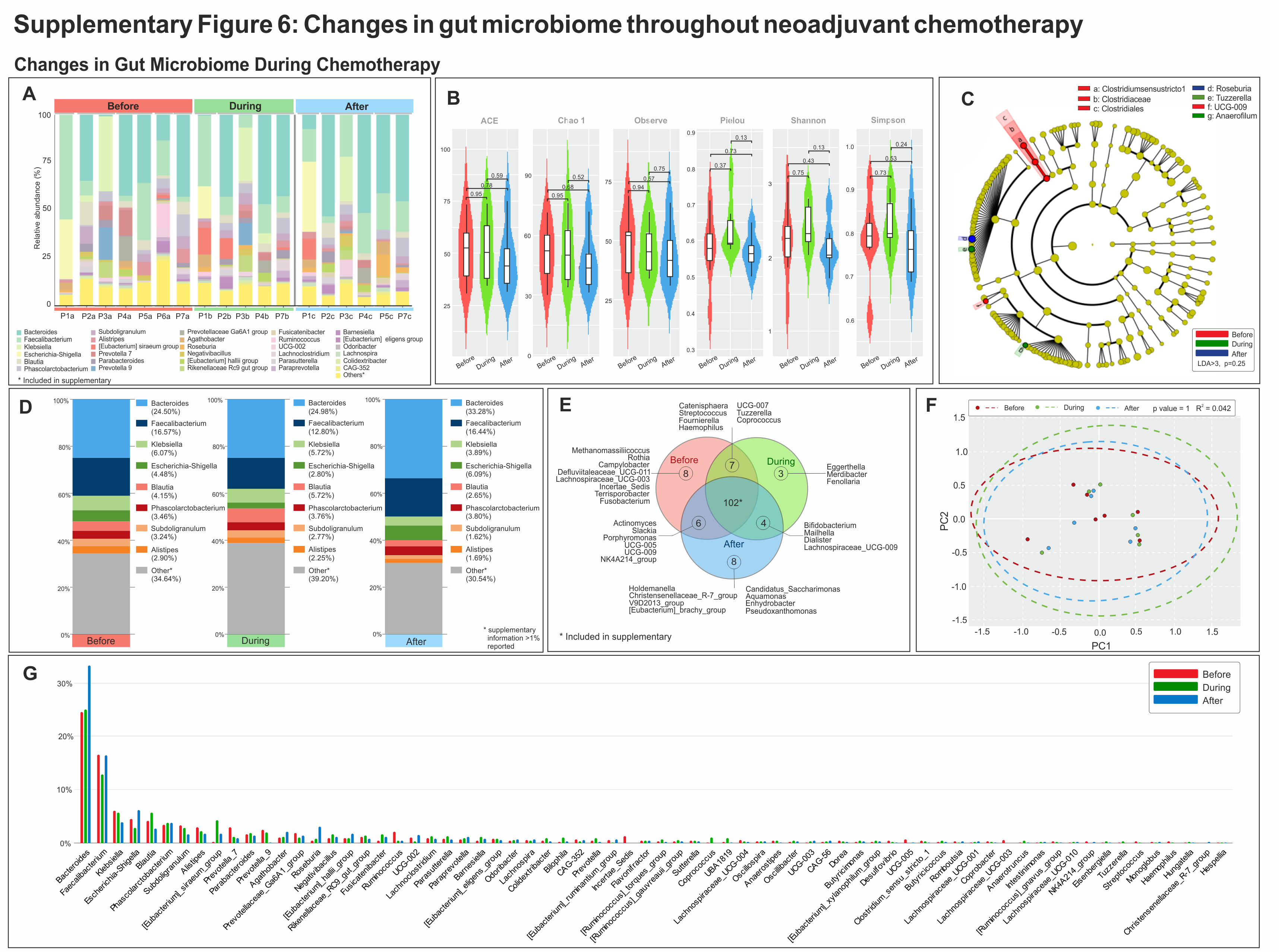

Supplement: Supplementary Figure 6 — Gut Microbiome Dynamics During Neoadjuvant Chemotherapy A: Relative abundance of major bacterial genera before, during, and after chemotherapy. Each color represents a different genus, with patient-specific changes highlighted to illustrate shifts in microbial composition over the course of treatment. B: Violin plots representing alpha diversity indices (ACE, Chao1, Observed, Pielou, Shannon, and Simpson) at three stages of chemotherapy: before, during, and after treatment. C: Cladogram derived from LEfSe analysis illustrating the most significantly altered taxa at each treatment stage. Colored nodes indicate taxa with statistically significant differences in abundance, with arrows highlighting key changes before, during, and after chemotherapy. D: Each segment shows the percentage composition of each genus by treatment stage. E: Venn diagram summarizing the overlap and unique bacterial taxa found at each stage of chemotherapy, quantifying shared and exclusive genera before, during, and after treatment. F: Principal Coordinates Analysis (PCoA) plot based on weighted UniFrac distances, showing the clustering of microbial communities at different treatment stages. G: Bar graph illustrating the proportional changes in specific bacterial genera over the course of chemotherapy, quantified and compared across three phases. [file crc-23-0479-s08.png]

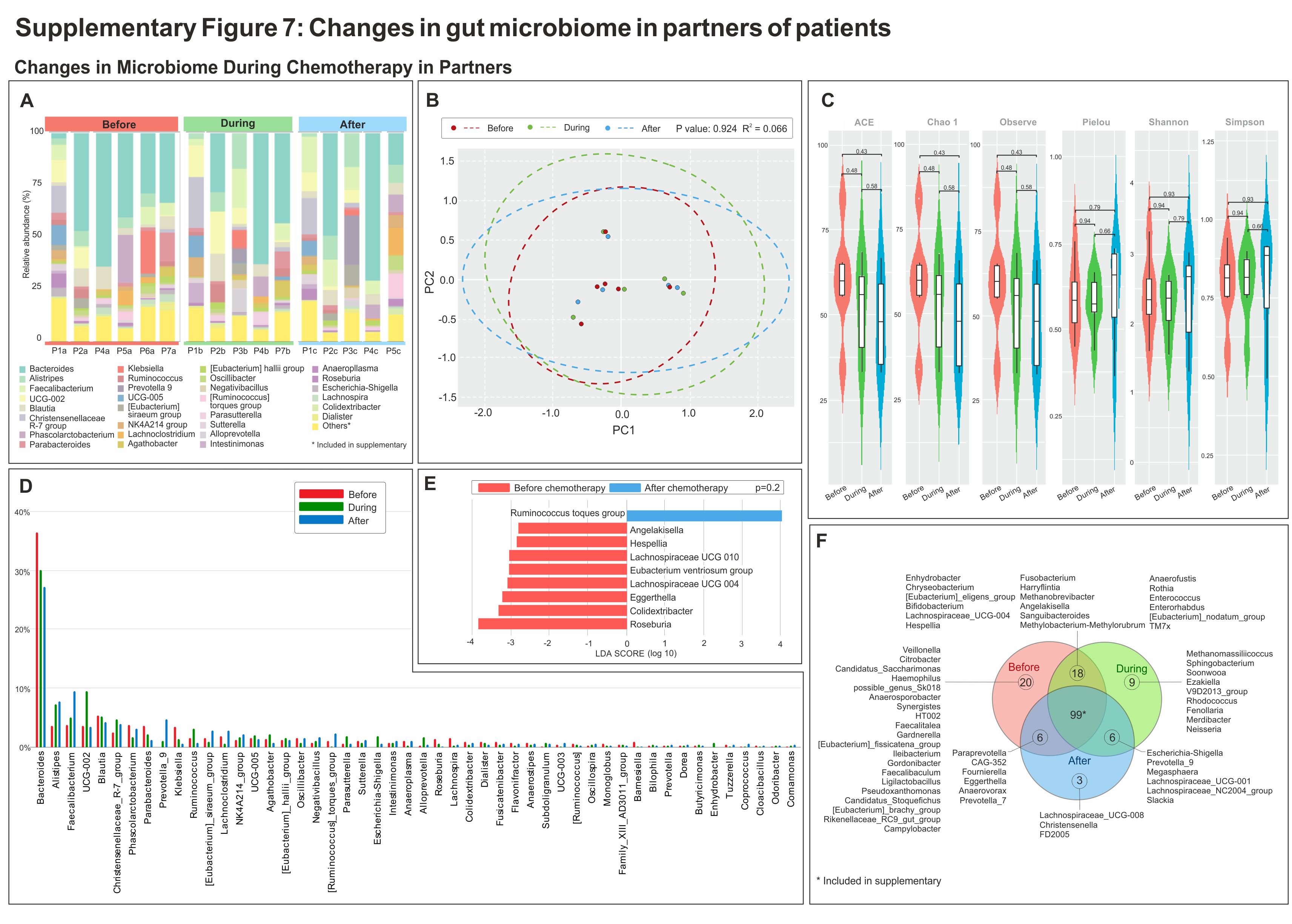

Supplement: Supplementary Figure 7 — Gut Microbiome Changes in Partners of Bladder Cancer Patients A: Stacked bar chart representing the relative abundances of key bacterial genera before, during, and after the chemotherapy periods in partners of patients. B: Principal Coordinates Analysis (PCoA) plot based on weighted UniFrac distances showing the clustering of microbiome samples at different stages of chemotherapy (before, during, after). The ellipses represent the 95% confidence intervals for each group. C: Violin plots illustrating the distribution of alpha diversity indices (ACE, Chao1, Observed, Pielou, Shannon, Simpson) at three key stages of chemotherapy. D: Bar graph depicting the percentage representation of selected bacterial genera across the three phases of chemotherapy in partners. E: Linear Discriminant Analysis (LDA) effect size (LEfSe) plot comparing bacterial taxa before and after chemotherapy, identifying which taxa are statistically significantly different between the two time points. F: Venn diagram illustrating the unique and shared bacterial taxa found at each phase of chemotherapy. [file crc-23-0479-s09.png]

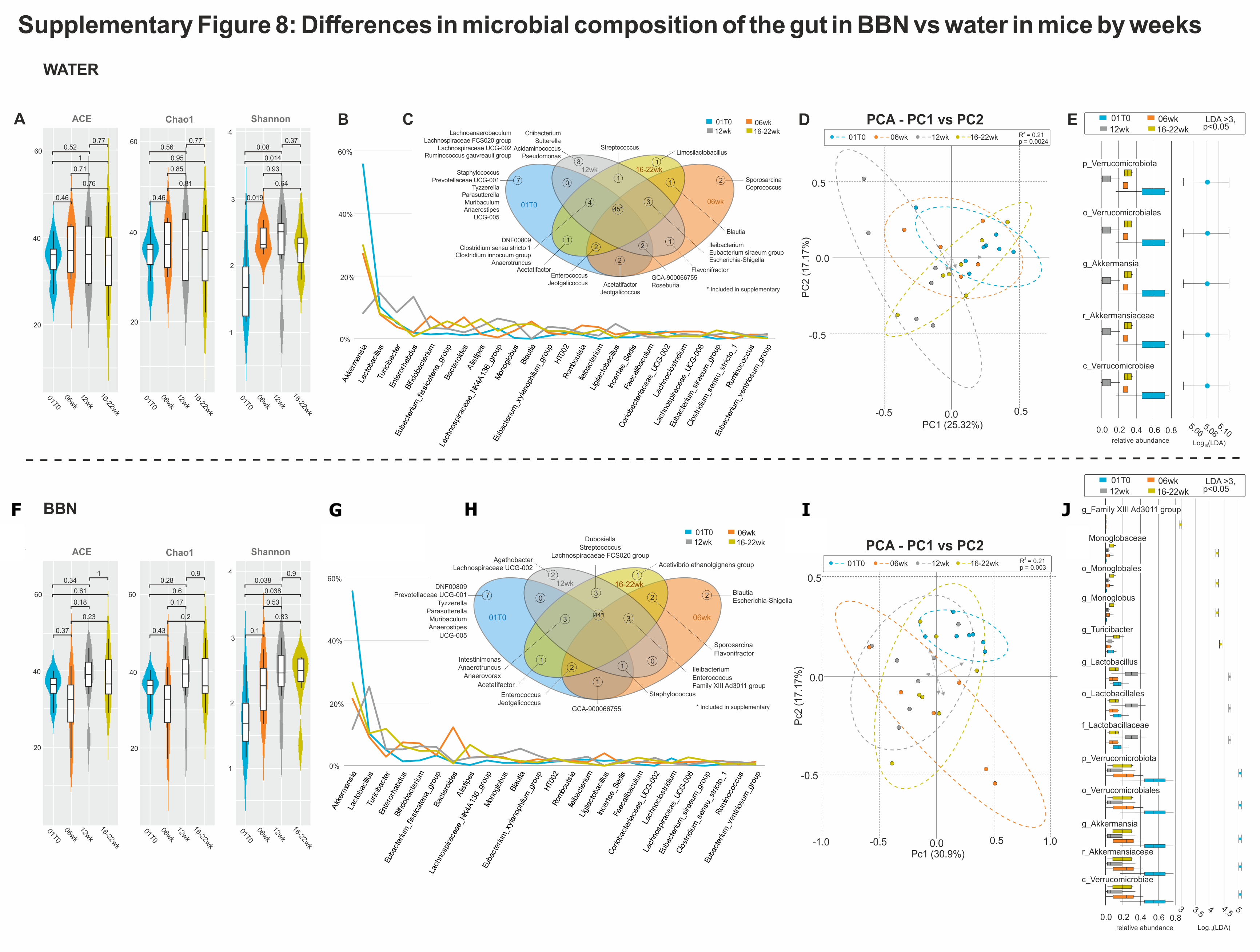

Supplement: Supplementary Figure 8 — Microbial Composition Differences in Mice Exposed to BBN Versus Water Water Group: A: Alpha diversity indices (ACE, Chao1, Shannon) plotted over multiple time points (0, 6, 12, 16-22 weeks), showing variations in microbial diversity in mice given water. B: Line graph depicting the percentage composition of bacterial families over time. C: Venn diagram highlighting the unique and shared bacterial operational taxonomic units (OTUs) at different time points. D: Principal Coordinates Analysis (PCoA) based on weighted UniFrac distances. E: Bar charts representing the relative abundance of specific taxa, identified as significantly different by LEfSe analysis, across the time points. The bars indicate changes in key microbial taxa that are statistically significant. BBN Group: F: Alpha diversity indices (ACE, Chao1, Shannon) for the BBN group. G: Line graph showing the dynamic changes in the percentage composition of bacterial families over time in the BBN-treated mice. H: Venn diagram detailing unique and shared bacterial OTUs between the different time points in the BBN group, illustrating the impact of the carcinogen on microbial turnover. I: PCoA plot for the BBN group, using weighted UniFrac distances to show clustering patterns that differ from the water group. J: LEfSe analysis bar charts showing the relative abundance of significantly different taxa across time points in the BBN group. [file crc-23-0479-s10.png]

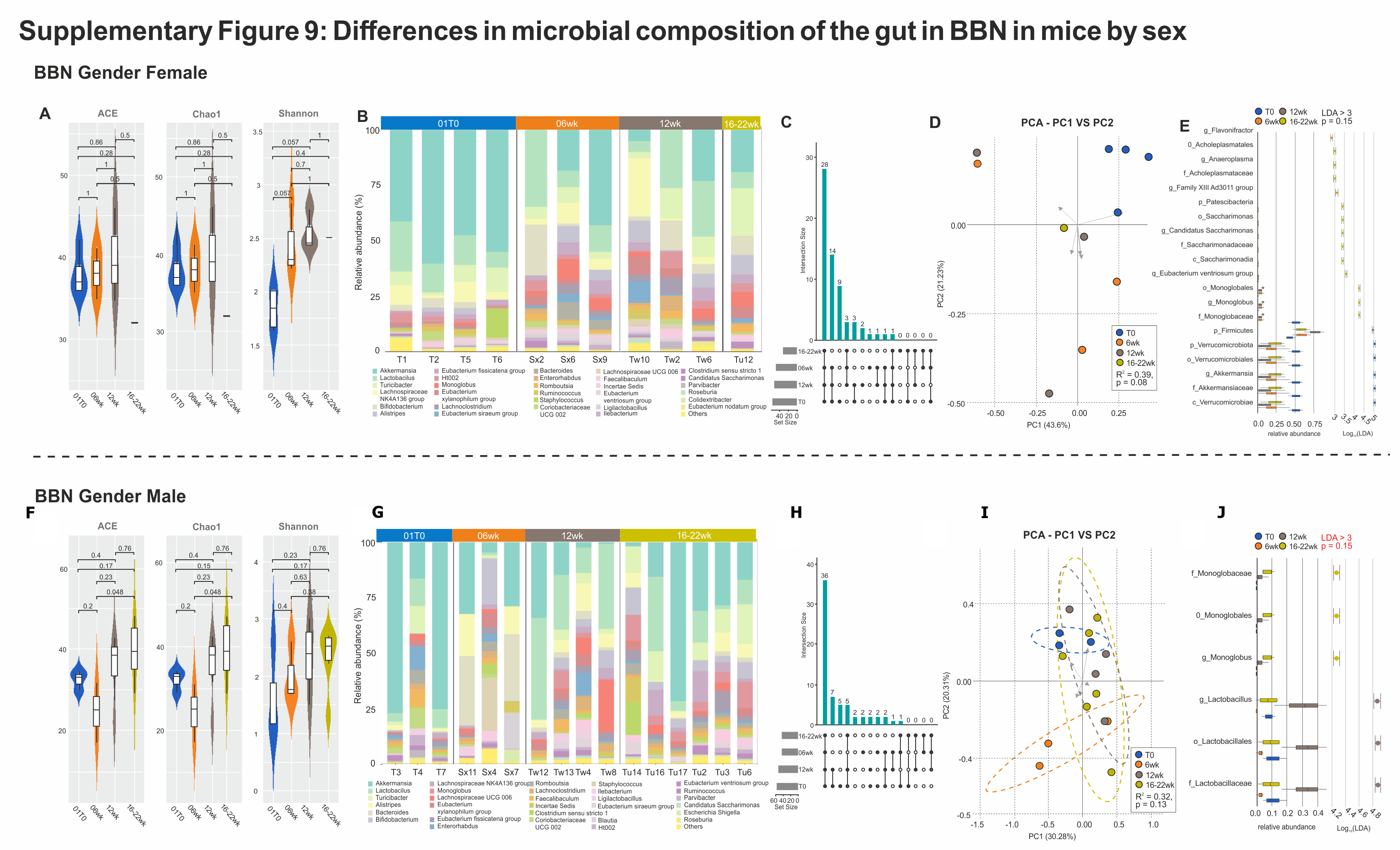

Supplement: Supplementary Figure 9 — Microbial Composition Variability by Sex in BBN-Exposed Mice BBN Gender Female: A: Alpha diversity indices (ACE, Chao1, Shannon) across different time points (0, 6, 12, 16-22 weeks), showing variations in microbial diversity in female mice. B: Stacked bar chart illustrating the relative abundance of bacterial genera at each time point. C: Dot plot depicting the percentage of specific bacterial families at different time points, providing a quantitative view of how predominant families fluctuate with BBN exposure. D: Principal Coordinates Analysis (PCoA) plot based on weighted UniFrac distances, showing the clustering of microbial communities across time, with color-coded points representing different time points to visualize the trajectory of community changes. E: Bar chart with Linear Discriminant Analysis (LDA) scores of bacterial taxa that are significantly different across the studied time points. BBN Gender Male: F: Alpha diversity indices (ACE, Chao1, Shannon) displayed across the same time points as the female group, illustrating the microbial diversity dynamics in male mice. G: Stacked bar chart showing the relative abundance of bacterial genera in male mice, and the specific microbial shifts unique to male physiology under BBN influence. H: Dot plot of the percentage representation of bacterial families over time, helps in identifying sex-specific microbial behavior in response to carcinogenic treatment. I: PCoA plot for the male group, illustrating shifts in microbial community structure with different clusters for each time point. J: LDA score bar chart for male mice displaying significant microbial taxa changes over time providing insight on impact of BBN exposure on the male gut microbiome. [file crc-23-0479-s11.png]
